# Self‐reported factors for improving patient's dental care: A pilot study

**DOI:** 10.1002/cre2.625

**Published:** 2022-07-01

**Authors:** Taina Kankaala, Pirjo Kaakinen, Vuokko Anttonen

**Affiliations:** ^1^ Research Unit of Population Health, Department of Cariology, Endodontology and Paediatric Dentistry University of Oulu 90014 Univeristy of Oulu Finland; ^2^ Dental Teaching Unit, City of Oulu Oulu Finland; ^3^ Research Unit of Nursing Science and Health Management University of Oulu Oulu Finland; ^4^ Medical Research Center Oulu University Hospital and University of Oulu Oulu Finland

**Keywords:** dental attendance, dental fear, inductive content analysis, pandemic

## Abstract

**Objectives:**

Patient‐centered approach can offer valuable information in improving dental care, but literature is scarce. This study aimed to evaluate self‐reported factors beneficial for attendance in dental care.

**Material and Methods:**

Survey data were collected during the summer of 2020 comprising both structured questionnaires and an open question on factors considered beneficial for dental attendance. Voluntary patients over 15 years of age (*n* = 196, 98%) agreed to fill the questionnaires, and *n* = 112 of them (57%) also gave open commentary in an urgent dental care clinic, City of Oulu, Finland, comprising the study population. Dental fear was assessed by Modified Dental Anxiety Scale (MDAS) sum scores (min 5, max 25). All utterances (*n* = 181) on an open question were evaluated by inductive content analysis to create sub‐ and main categories. Covid‐19 pandemic case counts in Finland were low at the time of the survey, but their effect on seeking dental care was asked. The distribution of patients was evaluated by cross‐tabulation, considering their age, gender, and dental fear status; the significance level was *p* < .05.

**Results:**

Females dominated slightly the study population (57%). The mean age of the respondents was 44 years. Half (50%) had moderate (MDAS score 10−18), and 10% had severe dental fear (≥19). The open responses could be categorized into four main categories. The largest main category by patient count concerned factors related to personnel (29%), followed by the patient (28%) and treatment (25%) related, and administrative factors (19%). Males chose factors falling into categories of administration and treatment while females chose patient and personnel‐related factors (*p* = .048). Compared to the rest, fearful persons (MDAS > 9) reported more often factors related to personnel and treatment (*p* = .03). Of all participants, 17% reported seeking less dental care during the pandemic.

**Conclusions:**

Patients value dental personnel and treatment‐related factors, specifically those with fear.

## 1 INTRODUCTION

Patient‐centered care (PCC) is one of the key elements in the literature on the quality of health services (Mills et al., 2014). In previous studies, especially in dentistry, the area of focus from a patient perspective has mainly been the quality of the outcomes of specific treatments, oral health‐related quality of life (OHRQoL), or patient satisfaction measured by structured questionnaires (Ali, 2016; Beecher et al., 2021; Conti & Humphris, 2019). Instead of patients themselves, patients' perspective on improving dental care has been estimated by healthcare professionals (Damiano et al., 2019). Although the concept of PCC is used and defined in various ways, the main idea is to keep patients always in focus. This requires skills, knowledge, and adequate resources of the staff (Pelzang, 2010). Studies on this topic in the public health care and general practice settings are still scarce (Mills et al., 2014). Modern dentistry aims to see patients as active members of the dental care treatment cascade (Anderson & Thomas, 2003; Chiou et al., 2020; Pelzang, 2010; Perazzo et al., 2020). Consequently, instead of listing barriers, patients could offer key elements for improving dental health care (Conti & Humphris, 2019; Krichauff et al., 2020). Vulnerable patient groups like dentally fearful patients can give valuable suggestions on how to improve their attendance and alleviate their anxiety during stressful dental appointments (Krichauff et al., 2020; Wang et al., 2017). A good way to activate the patients could be by encouraging them to give answers to open questions, and expressing their opinions and ways of thinking or feelings in their own words (Santha et al., 2015). The qualitative study can offer a deeper and wider perspective of the patient‐centered view given as responses to open questions when developing health care (Ravalika et al., 2019). Scientific evidence on the topic is only limited so far (Kyngäs et al., 2020).

The Covid‐19 pandemic has been reported to have had a strong impact on the healthcare system and healthcare workers as well as on patients' utilization of oral health services, access to regular dental care, and even urgent dental care (Guo et al., 2020; Jiang et al., 2020; Marcenes, 2020; Wu et al., 2021). Efforts have been made to ensure the safety of health workers as well as patients during dental appointments (Melo et al., 2021; Nardone et al., 2020; Villani et al., 2020). Oral health care is a part of the healthcare system, but oral health care has often not been recognized as an essential service when restrictions of the whole healthcare system have been applied during the pandemic (Benzian et al., 2021).

The aim of this mixed‐model survey (which offers both quantitative and qualitative data) was to evaluate factors reported by patients which could improve dental care and help patients utilize dental care services. In the analyses, inductive content analysis was used considering patients' age, gender, and dental fear. The impact of the Covid‐19 pandemic on self‐reported utilization of dental services was also studied. We hypothesized that a qualitative method may offer a new patient perspective needed in developing dental care. It was also hypothesized that the pandemic affects the utilization of dental services.

## 2 METHODS

### 2.1 Study population

The study material was collected during June and July 2020, representing both week and weekend days. Voluntary patients over 15 years of visiting dental urgent care (commonly patients have, i.e. cracks in teeth and pain, and they get preliminary treatment) in the City of Oulu Public Dental Services (PDS), Finland, were recruited to fill a semi‐structured questionnaire manually. The exclusion criteria for the original study population (*n* = 200) were: the immediate need for emergency treatment (*n* = 1) and general symptoms which required Covid‐19 testing (*n* = 1); two patients did not return the questionnaire.

### 2.2 Setting

Patients filled in the forms in the waiting area (*n* = 196, 98%). Of the respondents, 112 (57.1%) responded also in the open questions. The questionnaire was anonymous, only the patient's age (years) and gender (male/female/other/no answer) were asked as background information. The open question was as follows: “Which factors would make your dental visit easier?”. Patients were also inquired about the effect of the Covid‐19 pandemic on their use of dental care services as follows: *increased/decreased seeking dental care/no influence/can't tell*. Dental fear was evaluated by the Modified Dental Anxiety Scale (MDAS) (Humphris et al., 1995, 2000). The MDAS questionnaire has five questions with five different response options ranging from Score 1 representing not anxious at all to 5 extremely anxious (MDAS sum score min 5, max 25). In this study, we used three categories of the sum scores based on their distributions: 5−9 *low dental anxiety/*10−18 *moderate dental anxiety/*19−25 *high dental anxiety*. If a patient did not have enough time to fill the whole questionnaire before dental treatment began, the uncompleted forms were included as well. After responding to the forms, the patients placed them in sealed envelopes. Their ID number was written on the envelope. The first author (senior clinician) was available during the data collection if needed. A mixed model approach was used in analyzing the data when both qualitative and quantitative approaches were carried out.

### 2.3 Qualitative data analysis

**Figure 1:**
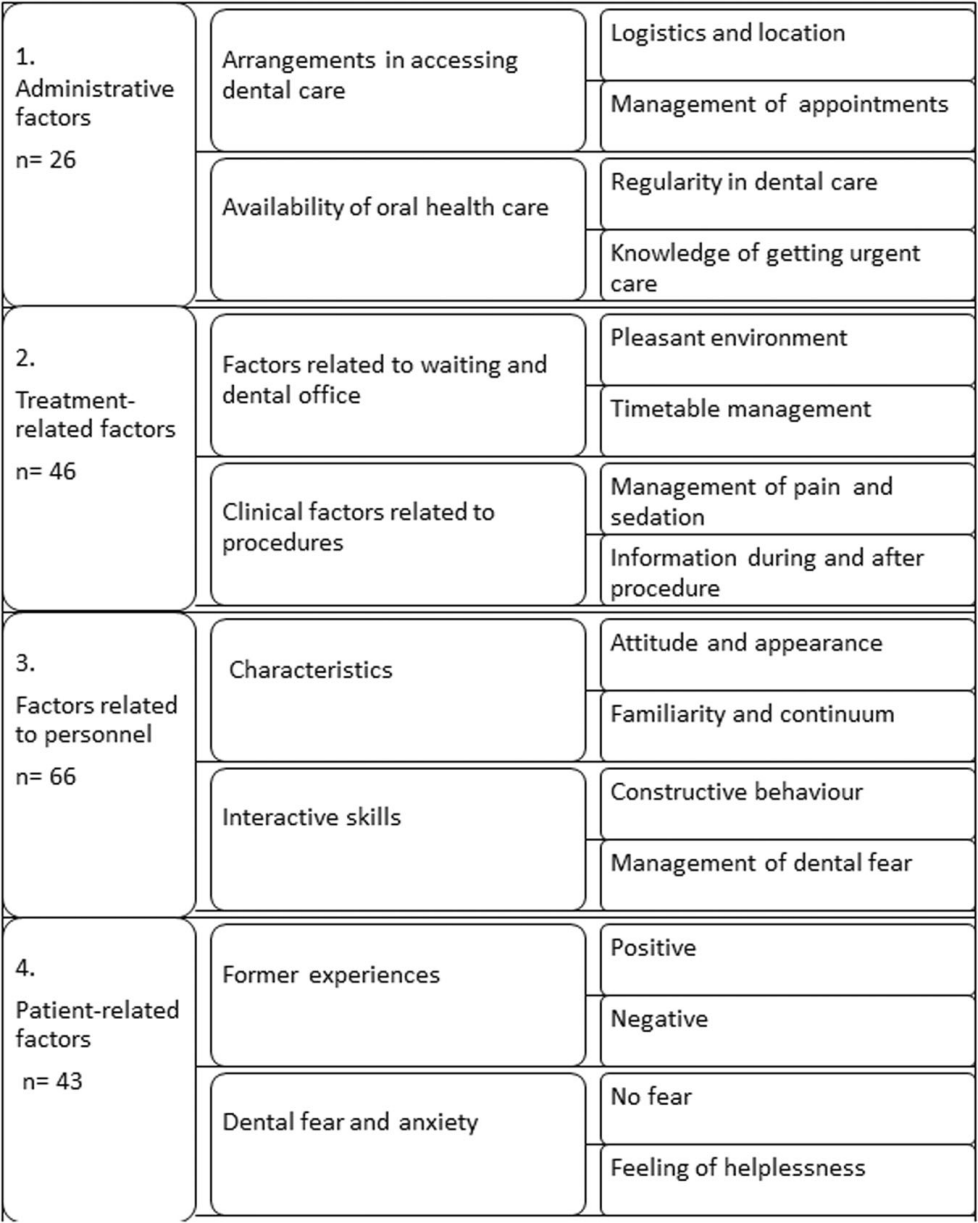
The process of inductive content analysis via subcategories and their descriptions. The number of utterances or expressions by the four main categories are included.

**Figure 2:**
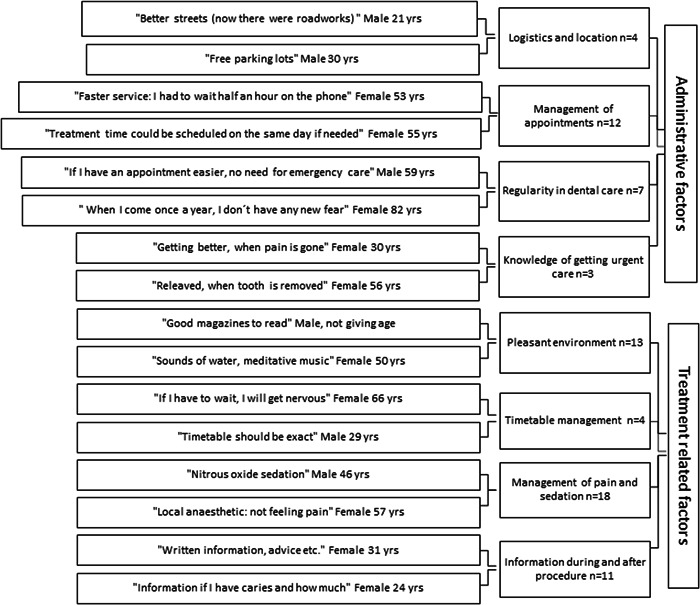
Examples of original utterances with their subcategories in main Categories 1 and 2: Administrative factors and treatment‐related factors.

**Figure 3:**
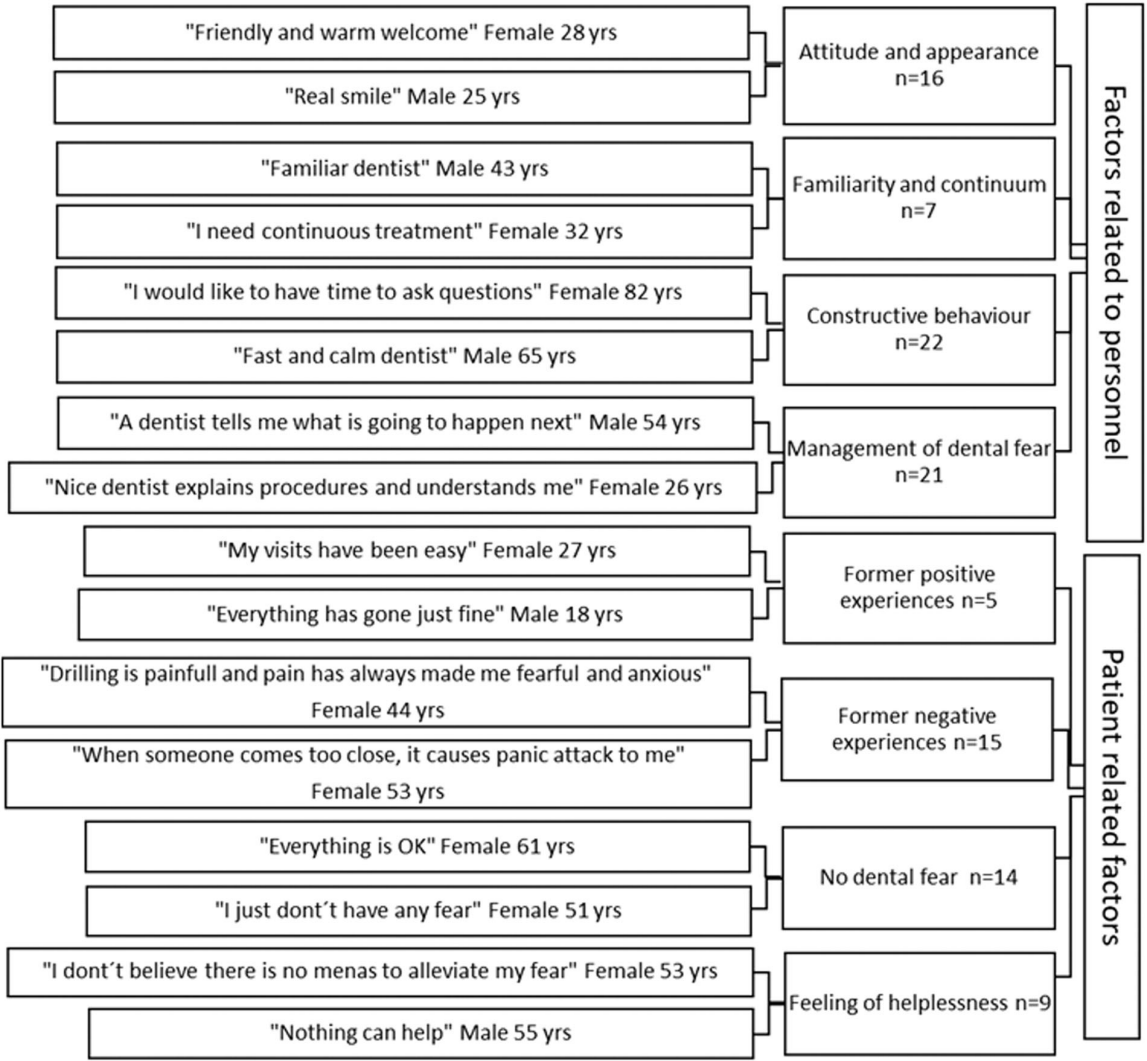
Examples of original utterances with their subcategories in Categories 3 and 4: Factors related to personnel and patient‐related factors.

Responses to the open question were analyzed using inductive content analysis (Kyngäs et al., 2000; Elo & Kyngäs, 2008) (Figures 1, 2, 3). Initially, open responses were either single words or longer utterances (*n* = 181). When open codes were generated from the responses, they were compared to find similarities and dissimilarities and organized into subcategories (Figures 2 and 3) by two authors (T. K. and V. A.) until replication was assessed and saturation achieved. Subcategories were combined into four larger main categories according to the content (Figure 1). A patient could give responses fulfilling the criteria of one or several subcategories leading to one or more main categories, but one category was recognized as the main category for each patient. If any part of the answer met the criteria of any other main category, those parts were encoded leading to the second, third, or even fourth category. The main categories were also investigated on a timeline of general dental treatment cascade (factors before and during a dental appointment, or both).

### 2.4 Statistics

The distribution of respondents and nonrespondents was carried out for age, gender, and dental fear (MDAS sum score and categorized sum score) using cross‐tabulation and the difference between the groups was evaluated by *χ* ^2^ test. The distribution of participants was analyzed by their main category, gender, age groups, and three MDAS score categories (*male/female/other*; ≤29 *years*/30−59 *years*/≤60 *years*; ≤9/10−18/≥19, respectively) using cross‐tabulation. The *χ* ^2^ and Fisher's exact tests were used to determine statistical significance at level *p* < .05. Quantitative analyses were performed, and graphs were designed using SPSS, version 26.0 (SPSS, Inc., Chicago, IL, USA).

### 2.5 Ethical considerations

Only voluntary patients over 15 years of age were recruited for the study. Written information was given to all patients and the study protocol was explained to them by the first author. All responses were analyzed anonymously, and all responses were given an ID. Answering the questions did not affect patients' dental treatment. A patient could refuse the use of his/her data in any phase of the study. Infection risk was carefully eliminated during the study and all instructions given by the local hospital district were followed. An ethics board statement was not required in this survey according to the Finnish National Board on Research Integrity (TENK) recommendation 2019 and this was verified by the Ethical Committee of Northern Ostrobothnia Hospital District in March 2020 before the study started. Data were collected with the permission of the City of Oulu (permission number §20/2020), Finland. The study was also registered under reference 140/2020 by Oulu University Hospital, Northern Ostrobothnia Hospital District, Finland.

## 3 RESULTS

The respondents were predominantly female (57%), and their mean age was 43.7 years (min 16, max 90, SD 16.7), 52% of them were between 29 and 50 years old. Only 3% did not give information about their gender and 2% about their age. Half of the respondents had at least moderate dental fear according to self‐reported MDAS score (MDAS > 9) and 1 in 10 were extremely fearful (MDAS ≥ 19). There was no significant difference between genders (*p* = .067) or MDAS sum scores among respondents and nonrespondents (*p* = .070), but those with severe fear were more likely than the rest to respond in the questionnaire (*p* = .009). The same was true for those under 60 years compared to the oldest group (*p* = .037).

Of the participants, 57% gave at least one suggestion on factors that would make their dental visits easier. They used an utterance or single words (Categories 1−3) for improving dental care when some described their former dental treatment experiences without any real proposals (Category 4) (Figure 1). Saturation was achieved after 51 patients in all subcategories and after 38 patients in all four main categories, but responses of all 112 participants were analyzed. Females gave more answers with multiple aspects which met the criteria of several categories compared to males. Almost one‐fourth of the patients gave additional secondary main category proposals, but only one patient (female, 82 years) gave proposals that met the criteria of all four categories (Table 1).

**Table 1:** Distribution of the participants in the four main categories (1−4) and three additional ones (second, third, and fourth) according to their responses

Number of participants, *n* (%)					
Main categories	Description of category	First category	Second category	Third category	Fourth category
1. Administrative factors	Arrangements and availability in accessing dental care	21 (18.8)	2 (1.8)	1 (0.9)	0 (0)
2. Treatment‐related factors	Factors related to waiting, dental office, and clinical procedures	28 (25.0)	13 (11.6)	1 (0.9)	0 (0)
3. Factors‐related to personnel	Characteristics and interactive skills	32 (28.6)	10 (8.9)	1 (0.9)	1 (0.9)
4. Patient‐related factors	Former experiences, dental fear, and anxiety	31 (27.7)	1 (0.9)	0 (0)	0 (0)
Number of patients, *n* (%)	Total *n* = 112 (100)	112 (100)	26 (23.2)	3 (2.7)	1 (0.9)

The inductive content analysis revealed factors in four main categories for helping make dental visits easier (Figure 1). Most of the original responses fell into the main category concerning personnel (Category 3), followed by proposals on the treatment (Category 2), factors related to the person him‐ or herself (Category 4), and administrative factors (Category 1) (Figure 1). Most of the original utterances on administration (Category 1) concerned appointments, location, logistics, whereas pain control, information during and after the appointment, and a pleasant environment were considered to improve dental treatment in the main Category 2 on treatment (Figure 2). A constructive, warm attitude of the personnel, as well as taking dental fear into consideration were reported to improve dental care in the main Category 3 (Figure 3). Category 4 comprised negative and positive experiences considered important by the respondents, previous negative ones even more so (Figure 3).

Males reported significantly more factors in the categories of administration and treatment (Categories 1 and 2), whereas personnel‐ and patient‐related factors (Categories 3 and 4) were more commonly reported by females (Table 2). The responses in different age groups did not vary significantly. However, the responses of the youngest age group tended to fall into Category 3 for dental personnel. Participants in the oldest age group provided wide narratives of their own experiences or fears (Category 4). The age group 30−59 years was the largest and their answers were evenly distributed between all four categories (Table 2).

**Table 2:** Distribution of the participants in the generic four main categories by age, gender, and MDAS‐scores

	Number of participants, *n* (%)				
Variable	Administrative factors	Treatment‐related factors	Factors related to personnel	Patient‐related factors	*p* value
Total (*n*)	21	28	32	31	
Age group					
16−29	5 (16.7)	4 (13.3)	13 (43.3)	8 (26.7)	
30−59	14 (24.1)	15 (25.9)	16 (27.6)	13 (22.4)	.095
60−90	2 (9.1)	8 (36.4)	3 (13.6)	9 (40.9)	
Missing	0	1	0	1	
Gender					
Male	12 (25.5)	15 (31.9)	8 (17.0)	12 (25.5)	
Female	8 (12.9)	13 (21.0)	24 (38.7)	17 (27.4)	.048
Other	0	0	0	0	
Missing	1	0	0	2	
MDAS‐score					
5−9	14 (31.1)	10 (22.2)	7 (15.6)	14 (31.1)	
10−18	7 (12.7)	16 (29.1)	20 (36.4)	12 (21.8)	.030*
19−25	0 (0)	2 (18.2)	5 (45.4)	4 (36.4)	
Missing	0	0	0	1	

* Fisher's exact.

Self‐reported fear measured by MDAS seemed to be associated with the responses (*p* < .030). Patients with little or no fear (MDAS score 5−9) gave answers focusing on administrative and patient‐related factors (Categories 1 and 4) whereas more fearful patients (MDAS > 9) focused their answers on treatment and personnel (2 and 3) (Table 2).

**Figure 4:**
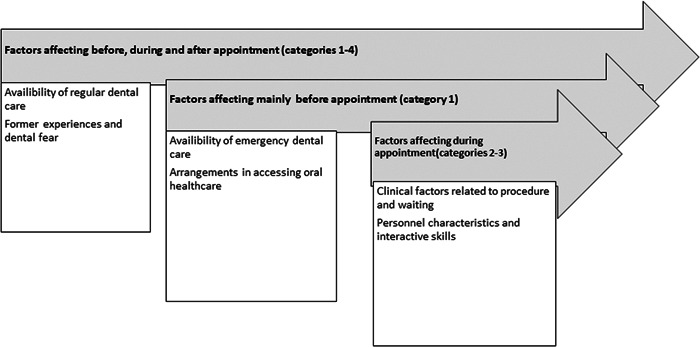
Main categories on the timeline of the cascade of dental care with examples of factors in the categories.

The logistics and availability of immediate urgent dental care were important factors before the appointment (subcategories of Category 1). The environment and timetable management in the dental clinic, factors associated with the procedures (subcategories of Category 2), and factors related to personnel (main Category 3) affected the experience during the treatment. Patient‐related factors (Category 4) as well as the availability of regular dental care (in a subcategory of Category 1) affected the experience of dental care in general (Figure 4).

One hundred and eleven (*n* = 111, 99%) of those who answered the open question also answered the question on Covid‐19. Two‐thirds (68%) reported that the pandemic had no effect on their utilization of dental services, 17% answered that the pandemic had decreased seeking dental care and 3% reported an increase.

## 4 DISCUSSION

This pilot study gives new and comprehensive information about patients' own expectations and suggestions for making dental care easier for them or improving dental care. Inductive content analysis proved to be a useful instrument for investigating this topic. The analyses revealed that suggestions for improving dental care were related to personnel, treatment, administration, and participants' own experiences. Males emphasized especially administrative‐ and treatment‐related factors such as concrete logistic solutions and pharmacological help for pain or fear, whereas females often considered the constructive attitude of the personnel important. Those who reported moderate or severe dental fear valued factors during the dental appointment or personnel‐ and treatment‐related factors. Arrangements to smooth access to oral health care and availability of urgent dental services are considered important factors preceding dental care, whereas treatment‐related factors and personnel characteristics are important during the appointment. Covid‐19 decreased the utilization of dental services among 17% of the participants.

The study population was large enough for qualitative analysis, to achieve full saturation and to have individual authentic multiple citations in all subcategories, which increased the reliability (Kyngäs et al., 2000). It was also large in comparison with recent qualitative studies in a dental care setting (Damiano et al., 2019; Gulion & Vergnes, 2019; Kurki et al., 2021; Wang et al., 2017). All participants were over 15 years or adults. As a new element compared to similar studies, here, patients could choose gender options other than male or female. This ensures that patients were not discriminated against by gender (Heima et al., 2017).

Not all responded in open commentary, the reasons for this can only be speculated. The analysis of respondents to open questions compared to nonrespondents showed no significant difference between the genders, whereas those under 60 years were more likely to respond compared to the older ones. The same was true for those reporting moderate or severe dental fear. This is interesting because, among all respondents, their distribution concerning dental fear was similar to the Finnish adult population (Liinavuori et al., 2016). So, it seems that fearful patients readily share their ideas, here mostly on personnel and treatment. The conclusion by Krichauff et al. (2020) and Wang et al. (2017) was thus confirmed. Yet, studies with bigger study populations in a general dental care setting are needed.

Today it is popular to do surveys online. Here the participants responded manually, so lack of access or lack of skills to use digital surveys did not affect this study sample. Our results are in line with those by Dolce et al. (2019) who reported that patients 75 years or older were more satisfied with dental care than the rest. For example, here two female patients (77 and 82 years) provided narratives including fearful memories but were very satisfied with modern dentistry: “Treatment has improved, more attention is paid to patients' sensitivity and fear of pain” and “New good instruments.” One in five of the participants was 60 years or older and they gave a variety of useful suggestions or were satisfied and grateful. For example: “I don't know, service is fine and the quality is high!” (Female, 65 years). The distribution of factors in different categories was similar between all age groups except that the oldest age group did not focus on the personnel as strongly as seemed to be the case among the youngest participants.

Inductive content analysis was selected as the main analysis method supported by quantitative analysis to offer new perspectives to improve PDS. Mixed analyses have been shown to be a valuable tool when information is scattered (Kyngäs et al., 2020) as it usually is when a patient‐centered view is investigated. Furthermore, abstraction and categorization offered means to establish factors emphasizing patient‐oriented future expectations on dental care. Previously, qualitative analysis using information about patients or their relatives has been shown to be valuable in finding ways to prevent adverse effects in medical care (Southwick et al., 2015). A patient‐centered qualitative approach can provide concrete, practical perspectives when new healthcare concepts are designed (Damiano et al., 2019; Gulion & Vergnes, 2019).

In responses, availability of dental care, both urgent and regular, as well as the continuum of care and a familiar dentist or staff, was repeatedly mentioned by the participants. This is new when previously, the main focus of interest has been on external factors, such as the costs of the treatment or adequate dental staff (Guay, 2004) or barriers in accessibility (da Rosa et al., 2020). Availability and accessibility were mentioned specifically by males who were appreciative of, for example, location and free parking. Only one patient mentioned that costs hindered her from seeking dental care (female, 82 years). However, even if it is a patient's choice to make an appointment or choose a pattern of avoidance, it must be kept in mind that many vulnerable patient groups may have these external or internal barriers to doing so (Gordon et al., 1998; Guay, 2004). This could most likely be improved by the suggested continuum and familiarity. Perhaps introducing low‐cost phone consultations with flexible, fast, and easy management of appointments or modern digital services will be a solution for low‐cost dental services in the future. The same can be true for walk‐in dental care (Gulion & Vergnes, 2019) and teledentistry (Daniel & Kumar, 2014).

Treatment‐related factors or outcomes are usually the main focus of interest in questionnaires concerning the quality of dental treatment, oral health‐related quality of life (Fueki & Baba, 2017; Mittal et al., 2019; Neelakantan et al., 2020), or dental fear treatment (Armfield & Heaton, 2013; Wang et al., 2017). Here, treatment‐related factors comprise one main category. Suggestions for good pain management and sedation as well as a need for information on the procedures and a pleasant environment were emphasized. Especially males suggested sedation as their only wish for improving dental treatment; two of them even expressed a wish for dental general anesthesia (DGA) as the first choice for dental care. The need for comprehensive information was, indeed, mentioned as frequently as local anesthetics. Patients hoped to have both written guidelines and easy access to digital materials. In previous studies, healthcare personnel has been trusted to be a reliable source of information (Chen et al., 2018). Dental staff and scientists should thus create high‐quality and understandable material available by multiple media to compete with possible biased information offered by various social media platforms.

The pleasant environment was appreciated especially by female patients. They suggested that distractive elements such as music or the sounds of water and beautiful pictures of nature should be present. This is in line with the results by Diette et al. (2003), who concluded that nature sounds and sights may reduce operation‐related pain. The urgent care clinic in this study was situated in a new and modern building, which could be one of the reasons why the patients made so few comments on the environment or facilities.

Personnel‐related aspects and especially communication skills have been found to be of utmost importance in previous studies (Chiou et al., 2020; Mills et al., 2014). This is in concordance with our study, when personnel‐related factors were reported especially by females and fearful patients. Good communication, understanding, and empathy should be practiced in all dental care, not just to treat patients with special needs (Kulich et al., 2003). Communication is essential in treating young patients (Conti & Humphris, 2019). Most of the suggestions for improvements included some examples or aspects of two‐way dialog: “I like when the dentist tells what he/she is doing and asks me often if I am fine” (female, 36 years). The participants valued the understanding attitude of the dentist and his/her ability to listen to them. Friendliness was also mentioned several times. Males emphasized mostly reliability and professional skills and preferred a relaxed atmosphere. Many patients suggested that they should have the possibility to continue treatment with the same dentists and nurses to enhance trust.

Treatment‐ and personnel‐related factors were associated with dental care situations when placed on a timeline here. This is of utmost importance in fear‐arousing situations like during surgical operations and extractions and patients should be given ways to cope with those situations. In the long run, this leads to better outcomes in terms of operation time and pain control (Koga et al., 2017) and benefits the patient. Previous studies have indicated that merely informing the dentist about dental fear can reduce the patient's anxiety (Dailey et al., 2002). In this study, the dentists were not aware of the patients' MDAS scores but filling out the questionnaires might have influenced some fearful patients positively. This was not studied here. On the other hand, when the participants were asked to fill MDAS and answer the open question in the same context, some fearful patients gave answers reflecting only on their dental fear. It seems that this opportunity to express fear or anxiety and past dental experiences is important. Indeed, the free commentary might be useful for reporting dental fear as part of the dental fear treatment process. On the other hand, studies on merely how to improve dental care would be valuable.

The timing of this study was June−July 2020 during the Covid‐19 pandemic situation after the first wave when the incidence of the Covid‐19 cases in the area was low. Interestingly, none of the patients commented on the restrictions due to the pandemic or fear of infection‐related factors in dental care. None of the patients complained about the strict instructions to use hand disinfectant, and face masks in the waiting room and keep distance while visiting dental care, either. Infections orientated from health care could deteriorate patients' trust in the treatment and personnel (Southwick et al., 2015). However, here, infection control was a factor valued by patients, because no comments or suggestions for improvements were made.

During the pandemic situation‐related restrictions, often mostly urgent and emergency care were provided in public dental services (PDS) (Benzian et al., 2021; Marcenes, 2020). Males tend to seek dental care as emergency care per se (Currie et al., 2017) whereas females favor regular dental care (Astrøm et al., 2011). Females were overrepresented in this study sample, which may support the idea that during the pandemic they had a restricted opportunity for regular dental appointments and needed urgent care. Furthermore, dental care avoidance, which leads to a vicious circle of postponed dental visits, deteriorating oral health, and consequently need for emergency care is well documented among fearful patients (Berggren & Meynert, 1984; De Jongh et al., 2011; Dou et al., 2018). Therefore, an increased proportion of fearful patients in the study population was expected here due to Covid‐19. However, the distribution of participants with high dental fear was comparable with the general population (Astrøm et al., 2011; Liinavuori et al., 2016). Running a study like the present one rather than one in the general dental setting was the only possibility during the pandemic.

The challenge here was the limited time for filling in the questionnaires in the waiting area. Patients were offered an opportunity to complete their answers after the appointment, but if they did not, their answers were analyzed anyway. Because this survey took place in the urgent care clinic, some patients stated that dental pain weakened their ability to concentrate on answering, specifically on the open question. They were encouraged to answer even briefly to give the first intuitively processed factor (Evans, 2011), which they considered to be of most importance. Careful efforts were made not to lead or advise patients when they were answering the questionnaire. With these limitations, the open question not leading a respondent in any particular direction and a fairly large study population are the strengths of this study.

## 5 CONCLUSION

Three categories of factors for improving dental care were found by qualitative content analysis: administrative and treatment‐related factors as well as factors related to personnel. Despite the fact that the study population comprised more fearful patients than general populations, these results are indicative of factors to be utilized in developing dental care. Patients value the availability of services, convenient location with easy access and parking possibilities as well as good transportation and a pleasant environment. They also appreciate continuum in the treatment, good communication and capable and friendly staff, and pain control. Additionally, they appreciate the information given during the entire treatment cascade, which may be done by various media. These are indeed keystones for good dental care. Due to the pilot nature of this study generalization cannot be done, and it would be valuable to conduct a similar study with a larger study population in a general dental setting after the pandemic to get a patient perspective on the development of dental care.

## AUTHOR CONTRIBUTIONS

Taina Kankaala and Vuokko Anttonen have been involved in all phases of the work: study design, data collection, analyses, and reporting. Pirjo Kaakinen has been involved in drafting the manuscript. All authors reviewed the results and approved the final version of the manuscript.

## CONFLICT OF INTEREST

The authors declare no conflict of interest.

## Data Availability

The data are kept at the University of Oulu and are available on reasonable request directed to the authors.
